# The Genome Segments of Bluetongue Virus Differ in Copy Number in a Host-Specific Manner

**DOI:** 10.1128/JVI.01834-20

**Published:** 2020-12-09

**Authors:** Yannis Moreau, Patricia Gil, Antoni Exbrayat, Ignace Rakotoarivony, Emmanuel Bréard, Corinne Sailleau, Cyril Viarouge, Stephan Zientara, Giovanni Savini, Maria Goffredo, Giuseppe Mancini, Etienne Loire, Serafìn Gutierrez

**Affiliations:** a CIRAD, UMR ASTRE, Montpellier, France; b ASTRE, CIRAD, INRA, Univ Montpellier, Montpellier, France; c UMR1161 Virologie, ANSES, INRA, Ecole Nationale Vétérinaire d’Alfort, Université Paris-Est, Maisons-Alfort, France; d Istituto Zooprofilattico Sperimentale dell’Abruzzo e del Molise G. Caporale, Teramo, Italy; Cornell University

**Keywords:** bluetongue virus, gene copy number, population genetics, virus-host interactions

## Abstract

Genome segmentation is mainly thought to facilitate reassortment. Here, we show that segmentation can also allow differences in segment abundance in populations of bluetongue virus (BTV). BTV has a genome consisting in 10 segments, and its cycle primarily involves periodic alternation between ruminants and *Culicoides* biting midges. We have developed a reverse transcription-quantitative PCR (RT-qPCR) approach to quantify each segment in wild BTV populations sampled in both ruminants and midges during an epizootic. Segment frequencies deviated from equimolarity in all hosts. Interestingly, segment frequencies were reproducible and distinct between ruminants and biting midges. Beyond a putative regulatory role in virus expression, this phenomenon could lead to different evolution rates between segments.

**IMPORTANCE** The variation in viral gene frequencies remains a largely unexplored aspect of within-host genetics. This phenomenon is often considered to be specific to multipartite viruses. Multipartite viruses have segmented genomes, but in contrast to segmented viruses, their segments are each encapsidated alone in a virion. A main hypothesis explaining the evolution of multipartism is that, compared to segmented viruses, it facilitates the regulation of segment abundancy, and the genes the segments carry, within a host. These differences in gene frequencies could allow for expression regulation. Here, we show that wild populations of a segmented virus, bluetongue virus (BTV), also present unequal segment frequencies. BTV cycles between ruminants and *Culicoides* biting midges. As expected from a role in expression regulation, segment frequencies tended to show specific values that differed between ruminants and midges. Our results expand previous knowledge on gene frequency variation and call for studies on its role and conservation beyond multipartite viruses.

## Abstract (precis)

The variation in viral gene frequencies remains a largely unexplored aspect of within-host genetics. This phenomenon is often considered to be specific to multipartite viruses. Multipartite viruses have segmented genomes, but in contrast to segmented viruses, their segments are each encapsidated alone in a virion. A main hypothesis explaining the evolution of multipartism is that, compared to segmented viruses, it facilitates the regulation of segment abundancy, and the genes the segments carry, within a host. These differences in gene frequencies could allow for expression regulation. Here, we show that wild populations of a segmented virus, bluetongue virus (BTV), also present unequal segment frequencies. BTV cycles between ruminants and *Culicoides* biting midges. As expected from a role in expression regulation, segment frequencies tended to show specific values that differed between ruminants and midges. Our results expand previous knowledge on gene frequency variation and call for studies on its role and conservation beyond multipartite viruses.

## INTRODUCTION

Variations in segment frequencies have been recently uncovered in within-host populations of multipartite viruses (1). Multipartite viruses are a diverse group of viruses, including DNA and RNA viruses, that infect mainly plants (2). Their genomes are composed of several segments, and in contrast to segmented viruses, each segment is encapsidated individually. Thus, each segment is thought to be physically independent from the others. Remarkably, certain segments are more abundant than others in within-host populations of these viruses (1, 3, 4). The term “genome formula” (GF) was thus coined to define the relative frequencies of all the segments in a within-host population. Moreover, segments tend to converge toward a specific frequency profile, the so-called “set-point GF” (i.e., the median segment frequencies in a population of infected hosts) (1). The fact that GFs deviate from segment equimolarity is puzzling from an evolutionary standpoint. Such population genetics should imply a cost derived from the higher probability of losing scarce segments during population bottlenecks (5). However, populations close to the set-point GF tend to accumulate at higher rates, suggesting a link with virus fitness (1, 4), probably through optimization of gene expression (1, 6). As expected from a role in expression regulation, the set-point GF has been shown to be host specific (1, 4). However, the putative relationship between GF and gene expression has not yet been directly demonstrated in multipartite viruses (7).

Currently, there are two examples of nonmultipartite viruses with a behavior reminiscent of set-point GFs in multipartite viruses. In the first example, populations of a monopartite baculovirus bore specific differences in gene frequencies that are due to certain genotypes with a deletion in the same genomic region (8). In the second example, a lower encapsidation rate of segment N led to unequal gene frequencies in populations of an experimentally evolved clone of influenza A virus, a segmented virus (9). In both cases, increases in fitness were observed despite the essential role of the genes with lower frequencies (9, 10). Moreover, fitness improvements were probably due to regulation of virus expression (9, 10).

These observations raise the question on how widespread gene frequency variation is. We have explored this question in bluetongue virus (BTV; *Orbivirus*, *Reoviridae*), a segmented virus affecting livestock worldwide. BTV possesses a double-stranded RNA (dsRNA) genome divided into 10 segments, a segment number among the highest in viruses. Most segments code for a single protein (11). Moreover, BTV is mainly an arthropod-borne virus infecting ruminants and *Culicoides* biting midges. This complex cycle probably involves yet-unknown mechanisms allowing expression regulation in phylogenetically distant hosts.

## RESULTS

We first built a collection of samples from naturally infected hosts during the 2014/2019 epizooty of BTV serotype 4 (BTV-4) in Europe (12). Blood samples were collected from sheep and cows from Corsica (France) and sheep from Sardinia (Italy), two Mediterranean islands (Table 1). The collection also included pools of 50 adult females of Culicoides imicola from the same islands and period (Table 1).

**TABLE 1:** Samples per host species and site^*a*^

Host	Island	Samples (no.)	Curated (no.)
Sheep	Corsica	24	11
Sheep	Sardinia	7	4
Midge	Corsica	39	5
Midge	Sardinia	10	4
Cow	Corsica	26	12

a Sheep, Ovis aries; cow, Bos taurus; midge, *Culicoides imicola*; Curated, samples in which RT-qPCRs of all segments provided a *C_T_* value below 31.

We then developed a SYBR green quantitative PCR (qPCR) approach to quantify genome segments similar to those used with multipartite viruses (1, 3, 4). The SYBR green assay was chosen over other qPCR assays due to its accuracy, simplicity, and cost efficiency in schemes requiring the validation of many primer pairs in parallel and the analysis of large sample numbers. Importantly, BTV segments can be quantified apart from viral transcripts because the negative strand of the dsRNA segments is generated only within fully formed capsids (13). Hence, we developed a two-step reverse transcription (RT)-qPCR assay for each segment of BTV-4 (strain BTV4‐16‐03) (12) in which the RT primer targets only the negative strand. We tested a large panel of primer couples to identify couples with similar efficiencies (minimum/maximum = 91.8/95.2) and specificity when using BTV-free templates (Table 2).

**TABLE 2:** Primers selected for the quantification of the negative strand of the BTV-4 segments

Segment	Direction	Sequence	5′ position	Amplicon size (bp)	Efficiency (%)	Mismatch^*a*^	Specificity threshold (*C_T_*)		Final concn (μM)
							Biting midges	Sheep blood	
1	Forward	AACATGGCTATTGGGACC	2213	125	91.9	>5	>39.5	>38.5	0.5
	Reverse	TCTGTAGTGTGTAGCTTTGTGTAA	2337						
2	Forward	TCTAGCTTCTCTATGTTTAGGGC	2541	70	93.0	>5	>39.8	>40	0.4
	Reverse	TTTCTTTGGATGCGCGAC	2610						
3	Forward	ATTCCGAGCGGCTTTAAGA	2287	75	94.5	>5	>40	>40	0.5
	Reverse	CGTACAGTGCGTAATACACC	2361						
4	Forward	TATGCGTAAGGGATTTGGTG	343	88	94.2	>5	>35	>35.3	0.5
	Reverse	GTTCAACGTCTCCGCTTC	430						
5	Forward	TGATCGCGGCAACTGAC	189	100	92.4	5	>40	>40	0.8
	Reverse	CAGACTGTTTCCCGATCATAC	279						
6	Forward	GTGAATCTTATGGAGAGTCGG	212	70	91.8	>5	>37.4	>40	0.5
	Reverse	GGTAATTCTTCACCTGTACCCAA	281						
7	Forward	ATGGCTACGATTGGTGTACTA	278	74	95.2	>5	>38.4	>38.7	0.4
	Reverse	ACGCGAGCAATCTCATTC	351						
8	Forward	AAGAGATGATTCCGGGAACTA	608	78	91.8	>5	>34.2	>34.8	0.4
	Reverse	CCAGCTTCCACCTCCTTA	686						
9	Forward	GGAACCCAAAGAGGAAGACA	133	110	95.1	>5	>37.5	>36.4	0.6
	Reverse	CCACATCTGCATCTTTAGC	242						
10	Forward	AAGGCTGCATTCGCATCGTA	242	115	93.5	>5	>40	>40	0.5
	Reverse	AGCCTCCTAGGTCGCTTTTC	356						

a Number of positions differing from sequence of the segment positive strand.

Each RNA sample was screened with the 10 RT-qPCR assays. To ensure exact calibration of the 10 qPCRs, we generated and used a single plasmid as the template for all the standard curves. This plasmid harbors a concatenate of the 10 regions targeted by our RT-qPCR assays, thus ensuring exactly the same initial number of copies per amplicon. We selected for further analysis only those samples showing a threshold cycle (*C_T_*) value below 31 for all segments (approximately 1,000 copies). The final data set consisted of 15, 12, and 9 samples from sheep, cows, and biting midges, respectively (Table 1).

**FIG 1:**
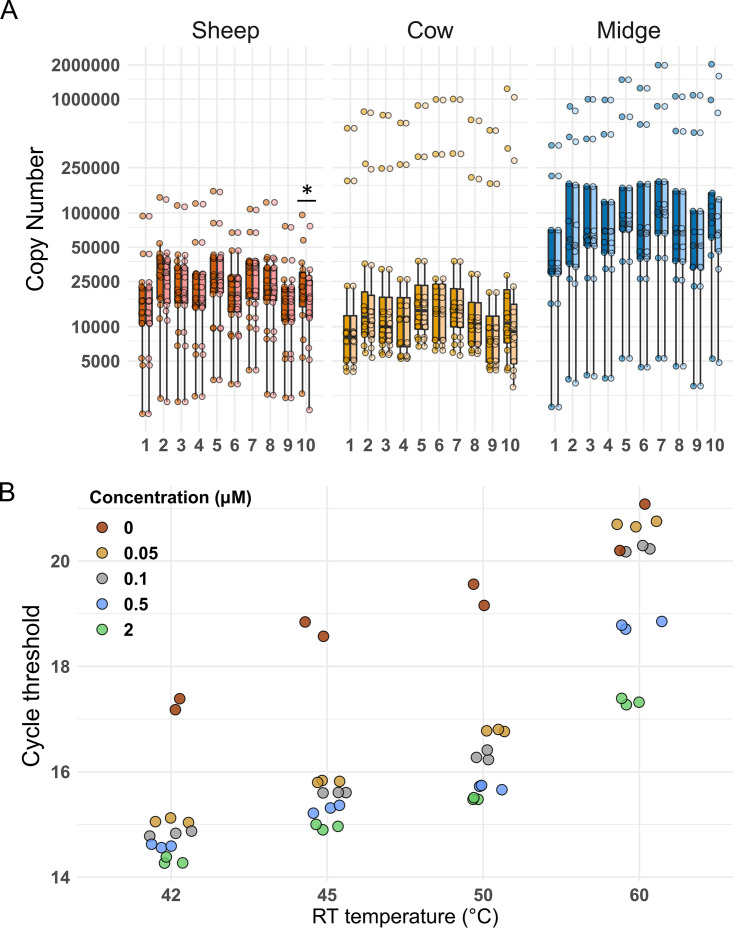
(A) Influence of primer-independent cDNA amplification on segment copy number (CN) in each host. CN values are colored according to host species, with lighter coloring for CN values after correction for primer-independent cDNA amplification. The correction consisted of subtracting the copy number yielded in an RT-qPCR assay without a primer in the RT step, and it was done for each segment and sample. Segment numbers appear at bottom. The asterisk indicates the only segment-host combination (segment 10 in sheep) for which a significant difference was detected between the uncorrected and corrected CNs. (B) Influence of temperature and primer concentration at the RT step on primer-independent cDNA amplification and qPCR sensibility. Data presented were obtained with the RT-qPCR assay for segment 10, the segment showing significant primer-independent amplification. RNA extracted from BTV-infected BHK cells was used as the template. RT-qPCRs were carried out as described in the text. Colors follow final primer concentration (μM) in RT step 1. Red dots indicate PCR output when RT was performed without a primer. The experiment was performed with two or three biological replicates. We selected an RT temperature of 42°C and a primer concentration of 2 μM.

**FIG 2:**
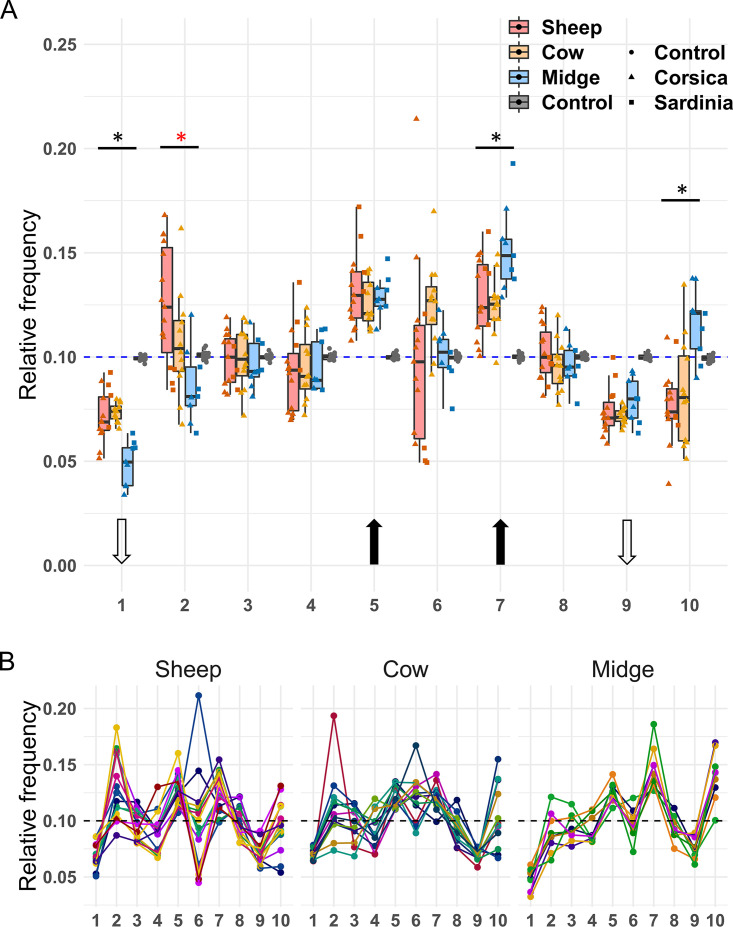
(A) Relative frequencies of BTV segments in naturally infected sheep, cows, and biting midges. The number of each segment is shown below the graph. The blue dashed line indicates the expected frequency under segment equimolarity. The results obtained with the plasmid control (“Control,” boxplots in gray) are shown to allow comparison with a template with equimolar amounts of PCR targets. Dot shape indicates sample origin (island) or template type (plasmid control versus field samples). The black and white arrows indicate the segments for which the average relative frequency was significantly higher or lower, respectively, than expected under equimolarity. Significant differences in frequencies among hosts for a given segment are indicated with a star above boxplots. Black asterisks correspond to significant differences between populations in the two ruminant hosts versus those in midges, whereas the red asterisk shows a significant difference between BTV populations from sheep and biting midges. (B) Segment frequencies per sample. Dots represent the relative frequency of a given segment (*x* axis) in a sample. Samples have been split per host in three panels (sheep, cow, and midge). For each host, all dots from a given sample are linked by a line of the same color.

We tested for primer-independent cDNA synthesis, a phenomenon in which RNA secondary structures or nucleic acid molecules (e.g., tRNA) can act as primers in the RT reaction (14). This phenomenon can affect strand-specific quantification in viruses (15), but to our knowledge, it is not tested for during quantification of BTV or other reoviruses (e.g., reference 16). We compared RT-qPCR output with or without a primer at the RT step for all segments and samples. RT reactions in the absence of primers for segments 2, 7, and 10 were the only ones yielding mean copy numbers at least 1% of that obtained with an RT primer (9%, 1%, and 26%, respectively; Fig. 1A). Primer-independent amplification could not be abolished without losing sensitivity through changes in the elongation temperature or the primer concentration at the RT step as previously proposed (15) (Fig. 1B). We compared segment frequencies in all samples between two data sets, corrected or not for the copy number obtained without a primer during the RT step. We observed a significant difference for segment 10 (Wilcoxon test, false-discovery rate [FDR] corrected, *P* value = 0.008), although this effect was detected only in sheep samples when the data set was split per host species (Wilcoxon test, FDR corrected, *P* value = 0.026 for the sheep data set) (Fig. 2A). Therefore, all statistical analyses were carried out in three data sets in parallel: (i) a data set with the raw copy numbers, (ii) a data set obtained after subtraction of copy numbers found in the absence of an RT primer (corrected data set), and (iii) a data set with raw copy numbers but without data from segment 10. No main difference in the significance of the statistical tests was observed between the three data sets (Table 3). The results presented below were obtained with the corrected data set.

**TABLE 3:** Analysis of factors influencing segment frequencies in three data sets^*a*^

Factor	*F* value			*P* value		
	Raw	Corrected	Wo-10	Raw	Corrected	Wo-10
Segment	52.16	56.77	68.00	<2e−16	<2e−16	<2e−16
Host	0	0	0	1.000	1.000	1.000
Island	0	0	0	1.000	1.000	1.000
Segment:host	6.18	6.21	5.62	6.51e−13	5.47e−13	1.71e−10
Segment:island	5.58	6.57	7.95	3.87e−7	1.38e−8	1.19e−9
Host:island	0	0	0	1.000	1.000	1.000
Segment:host:island	1.92	1.91	2.14	0.048	0.049	0.032

a Analyses of variance (model: frequency ∼ segment × host × island) with three data sets (raw, corrected, and Wo-10). Raw, raw copy numbers per segment and sample; corrected, copy numbers after subtraction of copy numbers found in primer-independent amplifications; Wo-10, raw copy numbers for all segments but segment 10.

**FIG 3:**
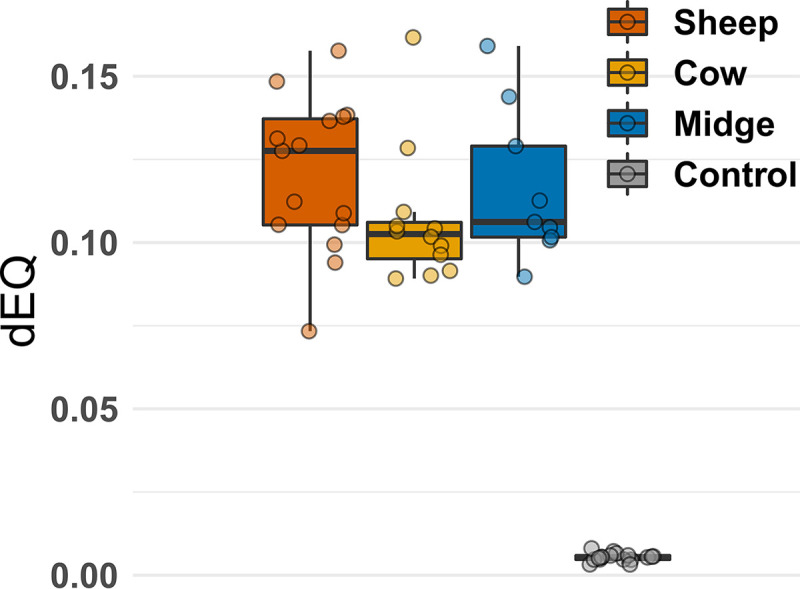
Distributions of segment frequencies per sample and host. Each dot indicates the distance to equimolarity of segment frequencies in a given sample (dEQ; see Materials and Methods for the formula). dEQ estimates obtained with the control plasmid (boxplot in gray) are provided to allow comparison with samples bearing equimolar amounts of PCR targets.

As observed in multipartite viruses, relative segment frequencies were variable and their distributions sometimes deviated from those expected under segment equimolarity (Fig. 2A). Moreover, distribution of segment frequencies in each sample did not suggest multimodality in frequency distributions between samples (for example, a group of samples with equimolar amounts of segments and another group with segment frequencies deviating from equimolarity) (Fig. 2B and Fig. 3). Segments 1 and 9 had lower frequencies and segments 5 and 7 had higher frequencies than expected under equimolarity in all hosts (Student’s *t* test one-sided, Benjamini-Hochberg corrected, *P* values <0.01) (Fig. 2A). The frequencies of segments 2, 6, and 10 also significantly deviated from equimolarity but only in sheep, cows, or midges, respectively (Table 4).

**TABLE 4:** Deviation from segment equimolarity^*a*^

Segment	Sheep		Cow		Midges	
	Less	Greater	Less	Greater	Less	Greater
1	5.31e−8	1	1.35e−9	1	1.32e−6	1
2	1	1.87e−3	1	0.295	0.106	1
3	0.293	1	0.431	1	0.117	1
4	7.34e−2	1	0.152	1	0.106	1
5	1	2.61e−5	1	4.88e−5	1	1.63e−4
6	0.571	1	1	9.58e−3	0.449	1
7	1	1.13e−4	1	9.57e−5	1	2.07e−4
8	0.589	1	0.152	1	0.106	1
9	3.97e−8	1	5.14e−10	1	2.09e−4	1
10	0.513	1	1	0.673	1	9.28e−4

a Relative segment frequencies were compared to the frequency expected under segment equimolarity (frequency = 0.1) using Student *t* tests (one-sided, correction with Benjamini-Hochberg method). The *P* values shaded in gray correspond to segments for which frequencies significantly deviated from equimolarity only in a single host (segments 2, 6, and 10).

An analysis of variance showed a significant effect of the variable “segment” on segment frequencies (Fig. 2A; Table 3). The analysis did not detect a significant effect of host or island but found significant interactions between segment and host, and between segment and island (Table 3). The analysis also found a marginally significant interaction among the three independent variables (*P* value = 0.049) (Table 3). Such crossover interactions are likely to take place when examining frequency data with several independent variables. We thus analyzed the effect of host species on segment frequency as previously done (1). Several factors hampered a robust analysis of a putative island effect, and it was not further explored (i.e., limited sample size, lack of cow samples from Sardinia, and differences in sample processing between islands).

No significant difference in the frequencies of any segment was found between BTV populations from ruminants (Tukey’s honestly significant difference [HSD] test for each segment with host as independent variable [Fig. 2A]; the full statistical output can be found at https://github.com/loire/MatSupp_Moreau20JVir). In contrast, segments 1, 7, and 10 showed significant differences in their frequencies between midge and ruminant samples (Fig. 2A). A significant difference was also identified for segment 2 between midge and sheep samples (Fig. 2A).

**FIG 4:**
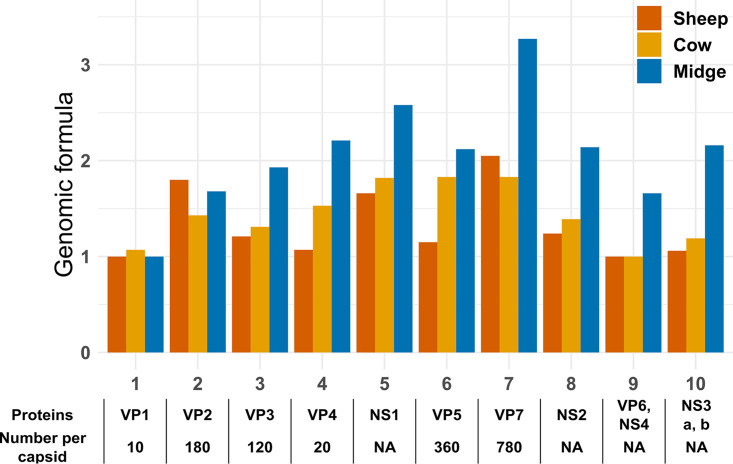
Set-point genome formulas are host specific in BTV-4. Set-point GFs were calculated for each host through dividing the median frequency of a given segment by the lowest median frequency among segments. The proteins encoded by each segment are shown below the graph (VP and NS stand for viral particle and nonstructural protein, respectively). Moreover, the stoichiometry in the capsid for structural proteins is provided when available (11). This information is provided to allow comparison between stoichiometry and set-point GFs.

These results supported the existence of host-specific set-point GFs in BTV-4 (Fig. 4). The relative abundances of segments 5, 6, 7, and 10 were between 2- and 3-fold higher than that of segment 1 in midges, whereas differences in ruminants were below 2-fold (Fig. 4). The largest differences were in the same order as those in nonmultipartite viruses with GFs improving fitness (between 3- and 4-fold) (8, 9).

## DISCUSSION

Deletions and nonconservative encapsidation have been observed in BTV populations infecting cell culture (17–19). These phenomena, undistinguishable with our PCR approach, could generate the observed differences among segments. Moreover, both phenomena would result in differences in the number of fully functional copies between segments and thus impact gene expression. However, if random, these phenomena alone cannot explain reproducible host-specific GFs. The observed GFs may also be (i) generated by host factors, (iii) encoded in the virus genome, or (iii) the product of selection on randomly generated GF variation (1, 5).

Host-specific GFs are expected under a hypothetical role in viral gene expression. However, we cannot provide a robust functional explanation on the differences between segments or hosts. For example, differences in copy number roughly followed stoichiometry in capsids for segment 7 (Fig. 4). However, this trend was perceptible only in midges whereas capsid structure is supposedly similar in ruminants and midges.

Whatever their role (or absence of), unbalanced GFs should have an impact on BTV evolution. If segments differ in their population sizes (i.e., their copy number), we could expect different evolutionary rates among segments and the genes they carry (5, 20–22). Moreover, a given segment could have different evolutionary rates depending on the host.

Overall, our results strongly suggest that wild BTV populations can have set-point GFs that differ from segment equimolarity and, even more intriguingly, between insect and ruminant hosts. This work expands our limited knowledge on gene frequency variation in viruses and calls for studies on its role and conservation in BTV and beyond.

## MATERIALS AND METHODS

### Sample collection and RNA extraction.

Samples were obtained from two Mediterranean islands, Corsica (France) and Sardinia (Italy), between 2016 and 2017. Blood samples from sheep were collected from symptomatic animals from farms undergoing a BTV outbreak in both Corsica and Sardinia. Blood samples from cows were obtained during routine testing for BTV in slaughterhouses in Corsica. Biting midges were collected using OVI traps as previously described (23), except that the water in the traps that was replaced with a solution limiting RNase activity (24). *Culicoides imicola* females were identified on a cold plate, gathered in pools of 50 individuals, and stored in absolute ethanol at −20°C for further investigation.

RNA extraction from Corsican samples was performed with the RNA viral extraction kit (Macherey-Nagel) following manufacturer’s instructions and using linear acrylamide (New England Biolabs) as carrier. Sardinian samples were extracted with the MagMAX core nucleic acid purification kit (Applied Biosystems) following manufacturer’s instructions.

### Development and validation of the quantitative PCR designs.

Primers were designed with the LightCycler probe design software 2.0 (Roche) using as the template the segment sequences of strain BTV4‐16‐03 (GenBank accession numbers KY654328 to KY654337). Primer pairs located in the central regions of segments were preferred. Primers for the reverse transcription of the complementary strand were tested for potential hybridization on the coding strand using Geneious v10 software, and only primers with at least 5 mismatches were selected (Table 2). Then, primer pairs underwent several experimental validations. First, primer pairs were tested for efficiency using serial dilutions of RNA suspensions from infected Vero cells as a template in the RT step. Dilutions were made in RNA suspensions from noninfected cell culture, to maintain RNA concentration constant. Primer pairs showing PCR efficiencies between 90% and 100% and a single peak during melting curve analysis of amplicons were selected for further validation. Specificity for selected primer pairs was tested against cDNA suspensions obtained from RT-processed RNA from BTV-free sheep blood and *Culicoides* biting midges and with concentrations similar to those of the field samples. Only primer pairs providing *C_T_* values above 34 were selected for further validation. Another round of efficiency validation was then carried out but this time using a plasmid as the template. The plasmid contained a concatenate of the regions targeted by primer pairs (i.e., each amplicon plus 20-bp flanking regions in both extremities of each amplicon), thus ensuring exactly the same initial number of copies per amplicon. Only primer pairs showing similar efficiencies and profiles of the melting temperature analyses between assays using either plasmids or cDNA were selected. Moreover, primer concentration was optimized to improve efficiency so as to obtain primer pairs with efficiencies at most differing in 5 units and, thus, with highly similar PCR dynamics. In total, we tested 61 primer pairs to generate the final set (Table 2; the primers rejected can be found at https://github.com/loire/MatSupp_Moreau20JVir).

### Reaction and cycling conditions of RT-qPCRs.

Tables 5 and 6 present the reaction and cycling conditions. First, RNA solutions were denatured with a dimethyl sulfoxide (DMSO) treatment. Then, RT and qPCRs were carried out in a two-step process. RT reactions were prepared separately for each segment as presented in Tables 5 and 6. RT reactions were performed with primers at a final concentration of 2 μM for all segments and as described in Table 5 with the Revert-Aid kit (Thermo Fisher Scientific) in a Simpliamp Thermal Cycler (Thermo Fisher Scientific). The RT primer volume was replaced with nuclease-free water in tests for primer-independent cDNA amplification. All qPCRs were carried out in triplicate (primer concentrations are shown in Table 2) with the LightCycler FastStart DNA Master Plus SYBR green I kit (Roche) in a LightCycler 480 thermocycler (Roche).

**TABLE 5:** Reaction conditions for the RT-qPCR assays: reagents and their volumes^*a*^

Reaction	Reagents	Vol (μl)
DMSO treatment	DMSO (100%)	2.8
	RNA template	11.2
		
RT step 1	Denatured RNA template	1
	Forward primer (20 μM)	1
	Nuclease-free water	10
		
RT step 2	5× reaction buffer	4
	RiboLock RNase inhibitor (20 U/μl)	1
	10 mM dNTP mix	2
	Revert-Aid RT (200 U/μl)	1
		
qPCR	Nuclease-free water	2
	Forward/reverse primers (10×)	1
	Master mix SYBR (2×)	5
	cDNA template	2

a Abbreviations: DMSO, dimethyl sulfoxide; dNTP, deoxynucleoside triphosphate.

**TABLE 6:** Cycling conditions for the RT-qPCR assays: temperature and length of each RT-qPCR step

Reaction	Length	Temp (°C)	No. of cycles	Step
Denaturation	3 min	95	1	DMSO treatment

RT step 1	5 min	65	1	Hybridization

RT step 2	60 min	42	1	Reverse transcription
	5 min	70	1	Reverse transcription

qPCR	10 min	95	1	Denaturation
	15 s	95	45	Amplification
	20 s	60	45	Amplification
	20 s	72	45	Amplification
	1 min	95	1	Melting curve
	1 min	58	1	Melting curve
	0.2°C/s	58–97	1	Melting curve

### Statistical analyses.

All statistical tests were carried out with the R software (R Development Core Team, 2011, version 1.2.5033). Distances of segment frequencies in a sample to equimolarity (dEQ) were calculated as $\text{dEQ }=\overset{10}{\underset{i}{\sum }}|p_{i}-0.1|/2$ according to the work of Manly (equation 5.7, p. 68 of reference 25), where *i* is the segment, *p* is the relative frequency of the segment in the sample, and 0.1 is the relative frequency at equimolarity.
